# iPad Use in Stroke Neuro-Rehabilitation

**DOI:** 10.3390/geriatrics2010002

**Published:** 2017-01-06

**Authors:** Khalid Ameer, Khalid Ali

**Affiliations:** 1 Brighton and Sussex Medical School, Brighton, BN2 5BE, UK; K.Ameer1@uni.bsms.ac.uk; 2 Academic Department of Geriatrics, Brighton and Sussex Medical School, Brighton, BN2 5BE, UK

**Keywords:** iPad, tablet computers, Stroke, neuro-rehabilitation

## Abstract

Neuro-rehabilitation services are essential in reducing post-stroke impairments, enhancing independence, and improving recovery in hospital and post-discharge. However these services are therapist-dependent and resource intensive. Patients’ disengagement and boredom in stroke units are common which adversely affect functional and psychological outcomes. Novel techniques such as use of iPads™ are increasingly researched to overcome such challenges. The aim of this review is to determine the feasibility, effectiveness, acceptability, and barriers to the use of iPads™ in stroke neuro-rehabilitation. Four databases and manual literature search were used to identify published studies using the terms “iPad”, “Stroke”, and “neuro-rehabilitation”. Studies were included in accordance with the review selection criteria. A total of 16 articles were included in the review. The majority of the studies focused on iPads use in speech and language therapy. Although of small scale, the studies highlighted that iPads are feasible, have the potential to improve rehabilitation outcomes, and can improve patient’s social isolation. Patients’ stroke severity and financial limitations are some of the barriers highlighted in this review. This review presents preliminary data supportive for the use of iPad technology in stroke neuro-rehabilitation. However, further research is needed to determine impact on rehabilitation goals acquisition, clinical efficacy, and cost-efficiency.

## 1. Introduction

More than 150,000 recognised cases of stroke are reported annually in the UK [1]. With more emphasis on primary and secondary prevention initiatives aimed at cardiovascular risk factors, stroke incidence rate has fallen by 19% from 1990 to 2010 [2]. Nevertheless, more than 1.2 million stroke survivors are living in the UK with long-term post-stroke impairments having a major impact on quality of life [2]. Such impairments range from motor weakness, visual problems, speech and swallowing difficulties, and psychiatric manifestations including anxiety and depression [3].

Neuro-rehabilitation services are an essential part of stroke treatment and recovery. These services help stroke survivors manage functional impairments, restore lost functions and regain independence. The National Institute for Health and Care Excellence (NICE) ‘Stroke rehabilitation in adults’ guideline highlights various recommendations for stroke rehabilitation services [4]. The gold standard recommendations include the provision of a 45 min therapy session from each member of the rehabilitation multi-disciplinary team for a minimum of 5 days per week. Despite such recommendations, there are several challenges that prevent the delivery of effective and adequate rehabilitation of stroke survivors. This is evident by the Sentinel Stroke National Audit Programme (2014) report highlighting that most patients do not achieve the recommended intensity and duration of rehabilitation as set by NICE guidelines [5]. Reported challenges to effective rehabilitation can be broadly classified into patient or system related factors [6,7]. Patient related factors include stroke severity, engagement, participation, boredom levels, and medical co-morbidities. While system related factors primarily include staff availability for the provision of NICE-recommended rehabilitation intensity. We know that rehabilitation is time-consuming, therapist-dependent, and resource-intensive. In view of these challenges in conventional rehabilitation programmes, there is a new call for novel strategies including use of tablet computers, gaming technology, and virtual reality.

In the modern era of technology, human-computer interface and innovative techniques in healthcare delivery have substantially increased over the last 10 years. The World Health Organisation (WHO) survey on eHealth view mobile technologies as having the potential in transforming health service delivery globally [8]. This is reflected by the increase in utilisation of commercially available tablet computers (e.g., Apple and Android tablets) in varying clinical settings including diabetic dietary management [9], stroke services (e.g., StrokePad™ for Stroke Assessments digital clinical records keeping in University College London Hospitals), and supporting shared decision making [10]. Lee Ventola (2014) comprehensively outlined the uses and benefits of mobile devices and software applications for health care professionals [11]. Such emphasis on technology is evident by the recent decision from the UK government to introduce free Wi-Fi internet connection in every NHS hospital by 2020 [12].

The wide touch screen platform, speech-to-text, and adaptive switches connectivity options in iPads allow for ease of use and navigation for stroke survivors. The visual-motor and somatosensory biofeedback provided by iPads allows for a more productive motor training and sensory stimulation optimising post-stroke neuroplasticity and cortical reorganisation [13]. Moreover, Apple™ offers the unique feature of creating “Apps for Healthcare Professionals” section within iTune Appstore encompassing diverse healthcare-related applications [14]. Such applications facilitate social interaction (e.g., Skype or FaceTime), recreational use (e.g., gaming applications) and therefore have the potential to reduce patients’ boredom and social isolation. Educational applications (e.g., StrokePatient™) provide stroke survivors with up-to-date and on-demand health information equivalent to traditionally used patients’ information leaflets. Overall, iPads have the advantage of being interactive, illustrative and easily accessible.

iPads provide a commercially widely available and relatively inexpensive form of technology (average cost depending on model start from £219). The telecommunications regulator Ofcom highlighted that over 54% of households own a tablet computer representing a 10% increase from 2014 [15]. The vast spread of this technology has transformed social interaction and overall lifestyle from browsing the Internet to reading the news and communicating with friends and family. It therefore arguably has the potential to benefit hospitalised patients and overall healthcare system [16]. With the aid of the widely available and easily accessible tablet computers, namely iPads (Apple Inc., Cupertino, CA, USA), stroke survivors can potentially benefit from longer duration of rehabilitation while maintaining their interest and engagement. Data from stroke survivors presented by Morris et al. (2010) highlighted that 90% of respondents own or have access to mobile device with 64% daily use and 80% consider it “very important” [17].

The devices are touch screen operated that react to the user finger movement once placed on the screen. Several components (e.g., gyroscopes) within iPads allow for the calculation and adjustments of orientation in response to users movement and tilting. This can be particularly useful in improving fine motor skills of stroke patients during upper limb exercises during occupational therapy sessions. Moreover, the option of forward and backward facing high-definition camera allows for diverse use within and outside therapy sessions. Therapist will be able to record patients’ sessions to provide real-time feedback and keep record of patients’ performance. The camera can also be used for social and recreational purposes to maintain contact with family and friends (e.g., Skype) and thereby reducing degree of boredom and social isolation that is prevalent in stroke unit [18,19]. Moreover, iPads allow for a diversity of use due to the wide range of applications for therapeutic, recreational, and educational purposes.

Various adaptive external switches can be connected to the iPads facilitating their use by patients with motor dysfunctions or challenges. The user is able to interact with the iPad and perform actions despite limited range of movement (e.g., eye blinking). Such adaptive switches can be particularly useful to aid communication of stroke survivors with speech and language impairments.

This review article aims to explore the feasibility, acceptability, benefits and barriers from the use of tablet computer technology, namely iPads, in the field of stroke neuro-rehabilitation.

## 2. Methods

### 2.1. Data Sources and Search Terms

A literature review conducted in August 2016 from 4 databases, including PubMed, Scopus, Cochrane Library, and Web of Knowledge. The following key search terms were used: (“Tablet computers” OR “iPad”) AND (“Rehabilitation” OR “Neuro-rehabilitation” OR “Stroke”). A manual search in major conferences, symposia, abstract databases and expert opinions was also conducted. Moreover, manual search of the references used by trials exploring tablet computers use but with different outcomes of interest to this review was also conducted to ensure no omission of relevant studies.

### 2.2. Selection Criteria

The following inclusion and exclusion criteria were used:

1. No date restrictions
2. Articles in English language only
3. Studies using iPad technology (Apple Inc. product) specifically and not generic tablet computer (e.g., Android), smart phones or PC
4. Stroke neuro-rehabilitation trials only

### 2.3. Data Extraction

A literature review is conducted using studies retrieved from journal databases and manual search. Only studies that met the selection criteria and suitability for the review question were selected. Article citations were imported electronically into EndNote X7 reference manager (Thomson Reuters, New York, NY, USA). A tabulation format on Microsoft Word 2010 (Microsoft Corporation, Redmond, WA, USA) was used to present extracted data from selected articles for the literature review. The data recorded included: author(s) names, year of publication, study type, number of participants, objective measured, and key outcomes reported.

### 2.4. Data Analysis

Studies included in the review were analysed to explore iPads use in stroke neuro-rehabilitation in term of: (1) feasibility; (2) impact on clinical outcomes; (3) impact on patients’ engagement; (4) impact of social isolation and boredom; (5) stroke survivors’ acceptability; and (6) role within community or home rehabilitation.

## 3. Results

### Database Search Results

**Figure 1:**
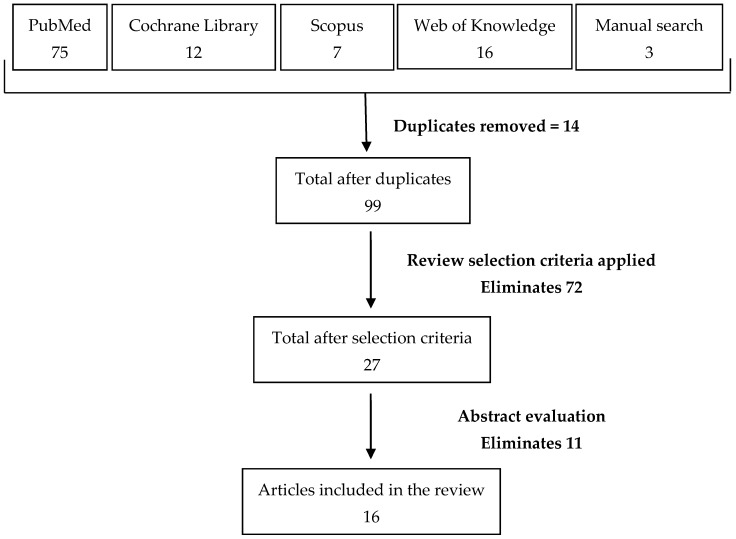
Flow diagram highlighting the number of articles found in each database and through manual search. A step-wise approach yielded a total of 16 studies to be included in this review.

A total of 104 articles were found in the database and manual search of which 14 were duplicates as illustrated in Figure 1. The review selection criteria eliminated 72 articles while abstract evaluation eliminated 11 further studies. A total of 16 eligible articles were included in this review. Table 1 provides a summary of the key studies and main outcomes presented in order of strength of hierarchy of evidence.

The studies were analysed to explore on the feasibility and clinical impact of iPad technology use in targeting post-stroke impairments in hospital-based and community rehabilitation. Emphasis on stroke survivors’ engagement and acceptability of iPad-based rehabilitation were also highlighted. Moreover, brief outline of the barriers to the use of iPads in stroke neuro-rehabilitation was also explored.

## 4. iPad Applications Used in Stroke Neuro-Rehabilitation

The Stroke Association UK recommends an extensive list of rehabilitation-specific applications, many of which are free of charge [32]. So far there are limited evidence evaluating the efficacy and drawbacks of the different applications for stroke neuro-rehabilitation. Current trials are underway in developing rehabilitation-specific iPad applications such as Stroke Rehab^®^ for home rehabilitation [33] and Rehab-let^®^ to improve dexterity and increase treatment intensity [34]. Table 2 summarises some of the literature on stroke rehabilitation specific gaming application using the iPad. It is important to note the variation in applications dependent on iPad device generation and iOperating Systems (iOS) version. Although currently undetermined, difference in iPad devices specifications within neuro-rehabilitation can potentially impact on user friendliness and effectiveness.

## 5. iPad Use in Post-Stroke Speech and Language Recovery

Although only few studies of small scales (number of participants ranged from 1–55) are currently available, they demonstrate the benefits of iPad technology in targeting post-stroke impairments and neuro-rehabilitation challenges. A lot of trials explored the use of stroke-specific iPad applications in speech and language therapy. iPad-based speech therapy applications are effective due to their relevance, personal nature, and applicability for repetitive task training. Therapists are able to record patients’ speech adding an element of biofeedback to enhance post stroke speech recovery. Studies were either conducted as a comparison of iPads to conventional neuro-rehabilitation programmes, or as an adjunct to existing therapy programme.

Although not specific to iPad technology, Brandenburg et al. (2013) review highlight the accessibility, potential uses, and challenges of mobile computing technology for the treatment of post-stroke aphasia [38]. Des Roches et al. (2014) highlighted the benefits of iPad-based software platform compared to conventional therapy in improving aphasia in stroke or traumatic brain injury patients [20]. The authors demonstrated improved language accuracy and latency (rhyming and syllable identification, word matching, and word problem) compared to control group who showed improvement in naming pictures only. The authors also demonstrated greater participation in repetitive tasks within experimental group than in control groups resulting in statistically significant improvement in Revised-Western Aphasia Battery and Cognitive Linguistic Quick Test scores pre and post therapy. Hoover and Carney (2014) demonstrated similar clinical benefits in the incorporation of iPads to provide comprehensive aphasia rehabilitation therapy [39]. Choi et al. (2016) recent pilot study highlighted positive findings on the effectiveness of iPad-based speech therapy application (iAphasia) for post-stroke chronic aphasia. The authors demonstrated significant improvement in language function as measured by the Korean version of Western Aphasia Battery score that was maintained during the 4-weeks follow-up [22].

Moreover, Lavoie et al. (2016) recent case study highlighted the effectiveness of self-administered iPad-based therapy for the treatment of post-stroke verb anomia in a 63 years old patient with chronic aphasia. The authors highlighted significant improvement in verb naming scores compared to baseline that was maintained during the 3 weeks follow-up visit [40]. Stark and Warburto (2016) pilot study highlighted similar positive findings of the effectiveness and feasibility of self-administered iPad speech therapy in chronic aphasia. The authors demonstrated significant improvement on expressive aphasia scores that was maintained in 50% (*n* = 5) of the participants during the 6 months follow-up visit [24].

## 6. iPad Use in Post-Stroke Motor Skills Recovery

In addition to improving therapeutic outcomes by means of better patients’ participation, tablet computers have the potential to benefit stroke rehabilitation by enhancing neuronal plasticity and rewiring [7,41]. Ample evidence demonstrates that neural plasticity following brain insults (e.g., stroke) is dependent on repetitive activity, intensity, frequency, and duration of therapy [13]. Tablet computers therefore have the potential to provide individualised and repetitive practice platform using an engaging medium. It allows patients to engage with a virtual dimension and thus increases neuronal activation in areas of the frontal, parietal, and temporal lobes [42]. This increased excitability has the potential in promoting cortical re-organisation and thus functional recovery while maintaining patients’ interest by virtue of exercise novelty.

Studies have demonstrated promising results in improvement of motor skills within physiotherapy and occupation therapy using iPads. Feedback from physiotherapists in our recent service evaluation study highlighted the benefit of iPads in recording stroke patients walking and transferring which were later used as learning and re-training tool [31]. Moreover, Kizony et al. (2016) examined the effectiveness of iPad-based application (“Tap-it”) on post-stroke hand dexterity impairment [29]. The authors demonstrated statistically significant (*p* = 0.0001) difference in finger tapping task performance in patients with post-stroke impairments and in healthy controls. As well as improved clinical outcomes, qualitative data highlighted positive overall experience from all patients participating in iPad-based rehabilitation. Nagayama et al. (2016) recently developed an iPad application (Decision-making in Occupation Choice (ADOC)) showing statistically significant (*p* = 0.027) improvement in Barthel Index in a single-blinded RCT for care-home elderly residents [43]. Rand et al. (2013) pilot study explored the feasibility and suitability of varying iPad applications in improving post-stroke hand impairment. The authors demonstrated a statistically significant correlation between hand weakness and improvement in hand performance using ‘Dexteria-Tap it’ and ‘Fast Tap’ applications [44]. The chief investigator is designing a stroke-rehabilitation specific iPad application (Rehab-let^®^) that target post-stroke motor recovery. A pilot randomised controlled trial exploring the effectiveness of this application is currently in the pipeline [34].

## 7. Impact of iPad on Patients’ Engagement, Social Isolation, and Boredom

Lack of meaningful activities and social isolation can be tedious and dispiriting for hospitalised patients in stroke units. This inevitably results in negative emotions such as depression, lethargy, and restlessness [18,19]. These negative sequelae can further prolong hospital stay that potentially be complicated by hospital-acquired infections, immobility, malnutrition, and lack of mental stimulation [45,46]. This is a particular challenge to hospital-based rehabilitation units due to significant findings of patients’ disengagement from meaningful or therapeutic activities [19,47]. A recent systematic review have highlighted consistent finding of large proportion of inactivity in stroke patients within the first 14 days of admission averaging at 65% of the day spent ‘inactive and alone’ [18,48]. This is concerning as conventional rehabilitation programmes are patient-dependent and are significantly limited by patients’ adherence.

Tablet computers can provide patients a form of entertainment through recreational applications (e.g., BBC iPlayer), facilitating communication with family and friends (e.g., FaceTime), as well as useful adjunct to targeted therapy. Utilising tablet computers therefore has the potential to reduce social isolation and might prove to be cost-effective by reducing length of hospital stay and services-dependency. Feedback from rehabilitation therapists in our recent service evaluation study indicated enhanced patients’ participation and engagement in therapy sessions with the use of tablet computers [31]. Therapists also reported reduced levels of patients’ abandonment of therapy sessions. As well as improved engagement, iPad interventions reduced patients’ feelings of isolation and boredom levels with 100% reported less boredom or between better and the same compared to baseline [31]. Moreover, a prospective pilot study using iPad technology on hospitalised (medical) patients’ showed increased patients engagement with high satisfaction due to ease of access to educational information and personal health records [49].

## 8. Patients’ Acceptability of iPad-Based Rehabilitation and Self-Management

In addition to improving functional recovery, several qualitative data demonstrates high acceptance and satisfaction of iPad-based rehabilitation programmes by stroke survivors [27,28,29,30,31,50]. Qualitative data presented by White et al. (2014) from 12 stroke survivors investigated stroke survivors’ acceptability of iPad-based rehabilitation in the first 3 months of recovery [30]. Through semi-structured interviews, the authors highlighted that patients found iPads easy to use with high acceptability, engaging and therapeutic [30]. Carabeo et al. (2014) indicated that patients recruited to the pilot study showed clear preference to tablet-based rehabilitation over conventional therapy [50]. Moreover, Rand et al. (2013) mean short feedback questionnaire (1–5 scale) showed high overall positive experience (4.5 ± 3.6 points) with iPad-based rehabilitation.

Our recent service evaluation study conducted at Sussex Rehabilitation Centre (SRC), Haywards Heath, showed similar findings with 89% of patients scoring either ‘satisfied’ or ‘very satisfied’ with iPad-based rehabilitation [31]. It was also noted that patients who have never previously used tablet computers were able to engage in the iPad-based therapy. Similar positive overall feedbacks were also received from staff members of the multi-disciplinary as they felt patients are more engaged in therapeutic activity. Nursing staff reported the benefit of iPad use for patients requiring 1:1 supervision as patients were found to be more settled and preoccupied due to recreational and social use of iPads [31].

Patients’ acceptance of iPad use in rehabilitation units demonstrates the potential for the device to be rolled out for community rehabilitation services. Patients’ recovery and progress can be monitored by the sharing of information electronically by tablet computers with the community rehabilitation teams [51]. Previous pilot studies of iPad-based home rehabilitation have shown high satisfaction rates as patients felt that the intervention is modern, engaging and cutting edge [30]. This allowed patients to continue their rehabilitation of similar intensity and further enhanced therapeutic gains post discharge.

Moreover, recent systematic review highlights the benefits of self-management programmes in empowering stroke survivors to take control of their rehabilitation, providing education, and enhancing patients’ adaptation to their post-stroke impairments [52]. Although yet to be determined, tablet computers can be used to support such programmes and facilitate sharing of information between patients and healthcare professionals.

## 9. Community Rehabilitation: Role of iPad

As per recommendations of NICE guidelines, stroke survivors receiving community rehabilitation services should be offered the same multi-disciplinary team input and intensity of therapy [53]. The Early Supportive Discharge scheme has shown promising results in reducing dependency, cost of service, and length of hospital stay [54]. Despite such scheme, several observation and service evaluation studies have highlighted the need for improved community rehabilitation services. A large stroke survivor’s survey conducted by the Wellcome Trust (2012) highlighted that only 41% of patients feel they are receiving enough support from NHS services after discharge [55].

As patients have easy access to iPads at home, some trials examined the feasibility and benefit of iPad-based home or community rehabilitation (Table 2) [27,33,56]. Kurland et al. (2014) studied the effectiveness of iPad use in home setting for enhancing and maintaining post-stroke aphasia recovery (object and verb naming) following a 2 week intensive speech and language therapy [56]. The authors demonstrated that following 6 months of home practice using the iPads, all 5 participants maintained their goals achieved during inpatient stay as well as learning new words. Moreover, Kiran et al. (2014) study on 55 stroke survivors demonstrated the benefit of iPad technology in facilitating continued post-stroke neuro-rehabilitation following patients’ discharge [21]. Speech therapists were able to track real-time patients’ progress during their follow-up clinic appointments and adjust intensity accordingly. Results from Saposnik et al. (2014) and Koh et al. (2016) RCTs will be of key interest in determining the feasibility and efficacy of iPads-based home rehabilitation [33,57].

Overall, preliminary results show promising evidence for the use of iPads in supporting patients’ discharge and ensuring continuity of rehabilitation within community setting. RCTs are still needed to assess the feasibility, clinical efficacy, and cost-effectiveness of iPad use within the community and home setting for stroke survivors.

## 10. Barriers to the Use of iPads in Stroke Neuro-Rehabilitation

Various barriers can be identified when considering iPad technology to stroke neuro-rehabilitation which can either be due to the nature of illness, the user (both stroke survivors and therapists) or the technology [38,58].

Post-stroke cognitive impairment, visual impairment, language deficits, and physical disabilities can be significant barrier to the interaction of stroke survivors with novel technology. Inability to understand and follow verbal or written instructions can impede both interaction with and functional benefits from conventional and alternative neuro-rehabilitation programmes. Motor skills deficits, particularly fine motor skills, can be a challenge when activating and navigating tablet devices. Moreover, stroke survivors’ acceptance, familiarity, and motivation to incorporate iPad technology to their rehabilitation programme can also be a limiting factor. Therefore, awareness of individual stroke survivor impairment is essential in ensuring patients-specific needs are targeted. This might include the use of speech-to-text options, external adaptive switches, font and graphics changes, and Augmentative and Alternative Communication (AAC) strategies [17].

Hospitals trust governance and healthcare services barriers are also an important aspect to consider when introducing tablet computers in hospital-based rehabilitation. Financial costs, logistics, security, securing Wi-Fi connectivity, and concerns over confidentiality are key limiting factors [58]. From our previous service evaluation study experience, we faced significant delay in implementing iPad use in inpatient rehabilitation unit due to governance approval issues relating to security and confidentiality [31]. Security issues have been adequately addressed by Apple Inc. in concordance with the US Federal Information Processing Standards (FIPS) 140-2 Level 1 rating iPads as very secure devices [59]. To ensure maximal patients’ data security and confidentiality, there is a need for setting up joint medical expert groups at local hospital and national levels between health trusts and commercial providers of tablet devices. Such data protection concerns are particularly relevant in potentially rolling out the use of iPad devises in future provision of tele-rehabilitation services for stroke survivors.

## 11. Conclusions

The incorporation of tablet computers to conventional rehabilitation post-stroke has the potential to maintain patients’ interest, improve clinical outcomes, and reduce abandonment of tasks. Few preliminary studies demonstrate positive results for iPad-based home rehabilitation. Further RCTs are still required to provide sufficient data on the effectiveness of iPads in enhancing stroke rehabilitation, adverse events, cost-effectiveness, and clinical impact on post-stroke impairments.

## Figures and Tables

**Table 1:** Review of the literature evaluating the use of iPad devices in stroke neuro-rehabilitation. Presented in order of hierarchy of evidence strength level.

Authors	Study Type	Participants	Objectives Measured	Outcomes
Des Roches et al. (2014) [20]	Case Control trial	51	Effectiveness of iPad-based therapy application (Constant Therapy) on aphasia in patients with stroke and traumatic brain injury	Increased language accuracy and latency Significant improvement in clinical outcomes pre and post therapy Greater patients participation
Kiran et al. (2014) [21]	Prospective Clinical efficacy study	55	Feasibility and efficacy of iPad-based software (Constant Therapy) in delivering continued and individualised post-stroke speech therapy (Home and clinic setting)	Improved task-specific accuracy and latency with iPad use Variable changes in language and cognitive measures Positive attitude from patients completing ‘homework practice’ using iPads following clinic therapy session iPads facilitated continued (long-term) rehabilitation for patients with chronic brain damage
Choi et al. (2016) [22]	Pilot study	8	Feasibility of iPad-base speech therapy application (iAphasia) for post-stroke chronic aphasia	Significant improvement in language function measured by Korean version of Western Aphasia Battery score Improvement was also noticeable during the 1-month follow-up Stroke survivors’ satisfaction was rated as ‘high’
McCormick and Holmes (2016) [23]	Pilot Study	13	Evaluating adherence, retention, usability, and adverse effects of iPad-based application (See, Imagine, Move; Upper Limb Action Therapy (SIMULATe)) for stroke survivors	iPads are feasible and acceptable intervention for post-stroke recovery More than 80% retention rate is reported No adverse effects reported Grip strength improved from 28.3 to 35.7
Stark and Warburton (2016) [24]	Pilot study and crossover design	10	Effectiveness and feasibility of self-delivered iPad-based speech therapy for post-stroke chronic aphasia	Significant improvement on expressive Comprehensive Aphasia Test (CAT) and Cookie Theft Picture Description (CTPD) Patients with lowest CAT baseline score made the greatest post-therapy improvement All patients were compliant with the recommended daily dosage of iPad use (20 min per day) 6 months follow-up on 5 participants showed that acquired improvements were maintained as measured by CTPD
Rand et al. (2013) [25]	Pilot study	22	Feasibility and suitability of iPads to improve post-stroke hand impairment	Control groups performance outweighed post-stroke group Statistically significant correlation between weak hand and hand performance as measured by The Nine Hole Peg Test, Fugl-Meyer Motor Assessment, and Box & Block Test using ‘Dexteria-Tap it’ and ‘Fast Tap’ applications No significant improvement for ‘Bowling’ application Short feedback questionnaire highlighted overall positive feedback from stroke patients using iPad-based rehabilitation
Kurland et al. (2014) [26]	Pilot study	8	Effectiveness of iPad use in home setting for chronic post-stroke aphasia following period of inpatient rehabilitation	All patients maintained speech and language goals obtained during inpatient rehabilitation following discharge Patients were able to continue daily SLT exercises and gain new words over 6 months period
Vandermaesen et al. (2014) [27]	Pilot study	5	Upper limbs motor improvement and motivation using tablet-based gaming application (ReHoblet) at home setting	Patients’ acceptance for the use of tablet computers as a rehabilitation tool Improvement in physical abilities and upper limbs motor function
Fizzotti et al. (2015) [28]	Proof-of-concept feasibility study	15	Feasibility of iPad use in rehabilitation	Improvement in trunk recovery scale score Integration with conventional rehabilitation is feasible Positive feedback from patients
Kizony et al. (2016) [29]	Proof-of-concept feasibility study	20	Feasibility of iPad application (Tap-it) in stroke rehabilitation	15/20 stroke patients were able to complete the trial No quantitative data presented Patients enjoyed the experience and felt that iPad use has the potential to improve hand function
White et al. (2014) [30]	Qualitative study	12	Stroke survivor’s perspectives on the use of iPad for therapy using semi structured interview	iPads easy to use Increased engagement and participation in therapy and recreational activities Feasible and acceptable
Suchak et al. (2016) [31]	Service evaluation	9	iPads feasibility, acceptance, and impact on patients’ boredom and social isolation in neuro-rehabilitation unit	iPad devices are feasible to be used in conjunction with conventional neuro-rehabilitation programme Acceptances from both patients and multi-disciplinary team Significant improvement in patients’ boredom and social isolation

**Table 2:** Some of the developed iPad applications that are incorporated with stroke neuro-rehabilitation. Clinical trials demonstrating efficacy still largely pending.

Application	Chief Investigator	Patient’s Impairment	Result of Trial
STROKE REHAB^®^	Saponsik et al. (2014) [33]	Fine motor weakness Neglect	Proof-of-concept RCT recruitment phase completed. Results pending.
REHAB-LET^®^	Rand et al. (2015) [34]	Upper limb weakness	Pilot RCT recruitment phase completed. Results pending.
iNeglect	Chung et al. (2013) [35]	Unilateral spatial neglect	Pilot study (*n* = 20) demonstrated feasibility of application to detect stroke patients with neglect and was helpful in objective assessment of improvement.
DEXTERIA-Tap it	BinaryLabs, Inc.	Fine motor skills	Statistically significant high correlation between the weak hand and hand performance as measured by The *Nine Hole Peg Test* (*p* = 0.037) and The *Box & Blocks Test* (*p* = 0.036) [25]
FRUIT NINJA	Bao X et al. (2013) [36]	Fine motor skills	Statistically significant (*p* < 0.05) improvement in Fugl-Meyer Assessment score and Wolf Motor Function Test score.
Constant Therapy	Constant Therapy Inc. (2013)	Speech and Language disorders	Improvement in language accuracy and processing speed [20]
VAST	Lindsley., (2009) [37]	Speech and Language disorders	Clinical trial pending
